# Knowledge, attitude, and practice towards hepatitis B virus among healthcare workers: a cross-sectional, hospital-based study in Montserrado County, Liberia

**DOI:** 10.11604/pamj.2023.46.77.27949

**Published:** 2023-11-09

**Authors:** Bluefin Masell Freeman, Sampson Chea, Bode Ireti Shobayo

**Affiliations:** 1 Department of Biological Sciences, College of Science and Technology, University of Liberia, Louisiana, Liberia,; 2 Department of Biology, Shaoxing College of Arts and Sciences, Shaoxing University, Zhejiang Province, China,; 3 Division of Public Health and Medical Research, Department of Technical Services, National Public Health Institute of Liberia, Monrovia, Liberia

**Keywords:** Hepatitis B, knowledge, attitude and practices (KAP), healthcare workers, Liberia

## Abstract

Prevention is critical in safeguarding against the spread of hepatitis B virus (HBV) infection. The knowledge, attitudes, and practices of healthcare workers (HCWs) regarding the disease play an important role in its prevention or spread, yet in this regard, data in Liberia remains scarce. Hence, determining the Knowledge, Attitude, and Practices (KAP) level of HCWs toward HBV infection becomes necessary. A descriptive cross-sectional hospital-based study was conducted among 251 HCWs at two major health facilities in Monrovia. KAP regarding HBV was assessed using a standardized structured questionnaire. Results were analyzed using descriptive statistics for demographic characteristics, percentages for categorical variables, and mean ± standard deviation for continuous variables. Kruskal Wallis test, p < 0.05, was used to derive statistical inferences. Complete data from 248 respondents showed a mean age of 36.3 ± 8.9 years with most respondents were within 30-39 age range (45.82%). While poor knowledge was observed in responses to all categories of questions, correct response rates to questions range from 41.93 - 99.19% for transmission of HBV, to 98.79 - 100% for preventive measures of HBV. About 77.8% and 90.32% of study respondents strongly agreed that hepatitis B is a major public health threat and that following infection control guidelines will protect them from being infected with HBV at work respectively. More than half of the participants (60.08%) had a history of needle-stick injury (NSI), and washing the injury site with water and soap, sterilizing the wound, and checking whether the patient has a blood-borne disease was done by 48.79%, 53.62% and 27.01% of the respondents respectively. Findings from this study show that there is an inadequate level of KAP regarding HBV infection among the HCWs. It therefore is expedient to conduct regular awareness campaigns for HCWs on preventive measures against HBV infection in hospitals, in addition to workshops and in-service trainings on infection prevention and control (IPC) best practices.

## Introduction

Hepatitis B virus (HBV) infection poses a serious global health burden resulting in significant morbidity and mortality from both acute infection and chronic complications [1]. A 2016 global burden of disease´s study reported that chronic hepatitis B infection (CHB) accounts for about 42% of deaths from liver cancer [2], with the greatest burden in Asia and sub-Saharan Africa accounting for 75% of cases of CHB worldwide [3]. It is estimated that 17.55 percent of Liberia´s 4 million people are estimated to be infected with hepatitis B [4].

Healthcare workers (HCWs) are at a significant risk of being exposed to blood-borne pathogens including viral diseases such as HBV [5]. A 2015 WHO global burden of the disease indicated that 37% of HBV infection among Healthcare workers (HCWs) resulted from sharp injuries sustained on the job [6].

The knowledge, attitudes, and practices of HCWs towards the HBV infection play an important role in its prevention or spread. This study sought to assess the knowledge, attitudes, and practices of HCWs regarding HBV, which is vital for targeted training and to build the capacity of HCWs to reduce the incidence of HBV infections and lower the burden of the disease in Liberia.

## Methods

**Study setting:** this descriptive cross-sectional hospital-based study was conducted between July and September 2017 in Monrovia, Montserrado County, Liberia. Two health facilities, one public (John F. Kennedy Medical Center) and one private (St. Joseph Catholic Hospital) were used for this study. These two major health facilities provide specialized inpatient and outpatient care in addition to being referral health facilities for a considerable proportion of the population in Monrovia. Monrovia, Liberia´s capital city is the most populated city in the country with a population of over 5 million [7].

**Study population:** the total of 251 healthcare workers including midwives, nurses, medical doctors, lab technologists, and hygienists working at the two participating hospitals were selected for this study using a non-probability sampling technique (purposive sampling). The criteria for inclusion were those willing to participate in the study while those refusing to participate in the study and those not in direct contact with patients were excluded.

**Study design:** data was collected using a self-administered questionnaire distributed at the participant´s work station. The questionnaire which was prepared in English consisted of four parts which focused on (a) demographic data (gender, educational qualification, marital status and occupational category), (b) knowledge of the HCW towards HBV infection, (c) attitudes of the HCW towards HBV infection, and (d) practices of HCW toward HBV infection as it relates to prevention of the HBV infection.

Each response on knowledge was labeled as ‘yes´ or ‘no´. Knowledge was scored by giving 1 for a correct answer and 0 for a wrong answer. The scale measured knowledge from a maximum of 13 to a minimum of 0. Scores ≥ 7 were taken as poor, > 7 as adequate knowledge of HBV.

**Statistical analysis:** descriptive statistics for demographic characteristics, percentages for categorical variables and mean ± standard deviation for continuous variables were used to analyze the results of the study. Kruskal Wallis test, p < 0.05, was used to derive statistical inferences and Spearman´s rank correlation coefficient (p < 0.01) was used to evaluate the association between KAP variables.

**Ethical considerations:** approval to conduct this study was given by authorities at the facilities where the research was conducted. Written consent was obtained from respondents before data collection.

## Results

Complete data was obtained from 248 study respondents with a 98.8% response rate and male: female ratio of 1: 1.43. Mean age was 36.3 ± 8.9 years (18-54 years). Poor knowledge was observed in responses to all categories of questions. Correct response rates to questions range from 41.93 - 99.19% for transmission of HBV (question 2), to 98.79 - 100% for preventive measures of HBV (question 4). More than three quarters (77.8%) and (90.32%) of study respondents strongly agreed that hepatitis B is a major public health threat and that following infection control guidelines will protect them from being infected with HBV at work respectively. Majority of respondents were considered to have a safe practice regarding HBV infection (60.53%), use of sterilized instruments (98.79%), and wearing of gloves (99.59%). More than half of the participants (60.08%) had a history of NSI. Only 35.88% of the respondents had completed the HBV vaccination (Table 1).

**Table 1:** knowledge, attitude and practices of healthcare workers regarding hepatitis B virus (HBV) infection (N=248)

Response to knowledge regarding HBV infection (N=248)									
**Questions**				**Yes (%)**				**No (%)**	
**Etiology of infection**									
Ever heard of HBV infection				248 (100%)				-	
Exposure to HBV infection can occur through prick from needles or sharp instruments				234 (94.35%)				14 (5.64%)	
HBV infection is caused by a virus organism				235 (94.75%)				13 (5.24%)	
Transmission									
Blood is infectious body fluid for HBV				246 (99.19%)				2 (0.80%)	
Vaginal fluid is infectious for HBV				109 (43.95%)				139 (56.04%)	
Amniotic fluid is infectious body fluid for HBV				191 (77.01%)				57 (22.98%)	
Urine is infectious body fluid				104 (41.93%)				144 (58.06%)	
**Symptoms**									
HBV can cause liver cancer				238 (95.96%)				10 (4.03%)	
Nausea, vomiting, and loss of appetite are common symptoms of HBV infection				196 (79.03%)				52 (20.96%)	
Jaundice is a common symptoms of HBV infection				243 (97.98%)				5 (2.01%)	
**Prevention**									
HBV infection can be prevented				248 (100%)				0 (0%)	
Vaccine is available for HBV infection				248 (100%)				0 (0%)	
After occupational exposure the HBV vaccine reduces the likelihood of being positive				245 (98.79%)				3 (1.20%)	
**Response to attitude regarding HBV infection (N=248)**									
**Questions**	**Strongly agree**		**Agree**		**Don’t know**	**Disagree**			**Strongly disagree**
HBV is serious public health problem	224 (77.8%)		15 (6.04%)		4 (1.61%)	2 (0.80%)			3 (1.20%)
All patients should be tested for HBV before they receive healthcare	49 (19.75%)		151 (60.88%)		12 (4.83%)	28 (11.29%)			8 (3.22%)
Being a health professional puts you at greatest risk of HBV infection	139 (56.04%)		90 (36.29%)		6 (2.41%)	11 (4.43%)			2 (0.80%)
Following infection control guidelines will protect me from being infected with HBV and HCV at work	224 (90.32%)		21 (8.46%)		0 (0%)	3 (1.20%)			0 (0%)
A healthcare worker can infect patients with HBV	77 (31.04%)		133 (53.62%)		16 (6.45%)	17 (6.85%)			5 (2.01)
Health professionals who are hepatitis B virus-positive should not give healthcare services to patients	28 (11.29%)		31 (12.50%)		11 (4.43%)	64 (25.80%)			114 (45.96%)
HBV vaccine should be compulsory	141 (56.85%)		47 (18.95%)		13 (5.24%)	32 (12.90%)			15 (6/04%)
Don’t need HBV vaccination because I’m not at risk	19 (7.66%)		38 (15.32%)		9 (3.62%)	126 (50.80%)			56 (22.58%)
**Response to practices regarding HBV infection (N=248)**									
**HBV practices**		**Number (correct answer)**					**Percent (correct answer)**		
Always sterilize instruments		245					98.79%		
Always wear gloves		247					99.59%		
Have been vaccinated completely for HBV		89					35.88%		
History of NSI		149					60.08%		
Wash hands with water and soap after NSI		121					48.79%		
Sterilized the wound site after NSI		133					53.62%		
Screened patient for blood-borne disease after NSI		67					27.01%		

HCV: hepatitis C virus; NSI: needle-stick injury

Among the demographic variables, age, education and profession were significantly associated with mean knowledge scores (p < 0.05). A significant difference was found for age where respondents in age group up to 20 - 29 years had a significant association with those in age group 30 - 39 and above 39 years of age with respect to knowledge on HBV (p-value - 0.0045). Similarly, for education and occupation, significant differences were observed. However, no significant association was observed as it relates to attitude and practices scores (Table 2).

**Table 2:** association between demographic characteristics and mean knowledge, attitude, and practices (KAP) scores for healthcare workers regarding hepatitis B virus (HBV) infection (N=248)

Variables	Number (n)	Knowledge score (mean ± SD)	P value	Attitude score (mean ±SD)	P value	Practice score (mean ± SD)	P value
**Age**			0.0045*		**0.615**		
Up to 25 years	95	15.14 (2.3)		8.29 (1.8)		2.97 (1.03)	0.0782
26-35 years	115	15.50 (2.8)		9.30 (2.4)		3.46 (1.1)	
Above 35 years	38	16.44 (2.2)		8.88 (2.1)		3.84 (1.2)	
**Education**							
Primary	7	16.39 (0.8)	<0.0001*	9.71 (2.2)	0.302	3.62 (1.2)	
Secondary	75	16.21 (2.6)		9.04 (2.1)		3.28 (1.1)	0.6166
Tertiary	166	14.12 (2.2)		8.73 (2.3)		3.37 (1.04)	
**Profession**							
Doctors	21	15.80 (2.7)	0.0226*	8.56 (1.8)	0.7553	3.26 (1.1)	
Nurses/midwives	204	16.71 (2.4)		9.15 (2.5)		3.35 (1.1)	0.1478
Laboratory technicians	23	16.62 (2.7)		9.52 (2.3)		3.52 (1.3)	

Kruskal Wallis Test (p< 0.05)

## Discussion

Results of the current study reveal that HCWs have an overall inadequate KAP regarding HBV infection. Knowledge that vaginal fluid and urine are infectious body fluids for HBV, and that nausea, vomiting, and loss of appetite are common symptoms of HBV infection are indicative that there is a need for more HBV health promotion, targeted education, and training of nurses and midwives. Other similar studies have also reported that the level of knowledge of hepatitis is low among different populations, including HCW, in several areas worldwide [8,9].

More than half of HCWS had been exposed to NSI and nearly the same number of them showed a negative attitude towards sterilizing the wound after the NSI and testing after exposure. A positive attitude was however shown by nearly all as it relates to sterilizing instruments before and after use and wearing of gloves. Although a majority of HCWs had knowledge of the availability of the HBV vaccine, there is still a need for concern because only a little over a quarter of them had been vaccinated completely. In developing countries, the gravity of such an issue as NSI tends to be underrated because many HCWs do not report exposure to hepatitis B virus infection or the risk of exposure. This could pose challenges for post-exposure management [10].

## Conclusion

Findings in this study suggest inadequate levels of KAP regarding HBV infection among the HCWs. This is concerning, especially since HCWs are a source of information to the patients they work with and may be passing down wrong information to them, in addition to influencing the attitudes and practices of patients towards HBV. It is therefore important that awareness campaigns on preventive measures against HBV infection especially in hospital settings should be conducted frequently among HCWs. Regular workshops and in-service training on IPC best practices should be conducted for HCWs.

**Limitations:** the scope of this study was limited by inadequate financial resources and time constraints. Sampling was also not by probability and therefore may not be representative of all HCWs in Liberia.

### What is known about this topic

- *HCWs are an important source of information to the patients as it relates to HBV infection and the prevention of its spread*;
- *Inadequate knowledge, poor attitude and bad practices by HCWs can significantly have a negative impact on the prevention of HBV infection*.

### What this study adds

- *The overall inadequate KAP of HCWs regarding HBV infection was inadequate*;
- *There is a pressing need for capacity building in IPC measures for HCWs in health facilities in Monrovia, Montserrado County*;
- *There is also a need for more HBV health promotion, targeted education, and training of HCWs*.
